# Policy Coordination or Fragmentation? A Content Analysis of Digital Aging Policies in China

**DOI:** 10.1093/geroni/igaf122.3394

**Published:** 2025-12-31

**Authors:** Sihan Lu, Lili Shang

**Affiliations:** Institute for Philanthropy Tsinghua University, Beijing, Beijing, China (People’s Republic); University of New South Wales, Sydney, Sydney, New South Wales, Australia

## Abstract

To address population aging, digital technology has become a crucial policy domain for improving aged care services and enhancing the well-being of older adults. The degree of policy coordination would significantly impact the implementation and effectiveness of digital initiatives. By synthesizing key digital aging policy documents from 2020-2024, this study aims to examine the degree of policy coordination, alignment of policy instruments as well as government organization in the Chinese policy landscape. Informed by one policy instrument framework, this study categorizes digital aging policies into supply-side, demand-side and environmental policy instruments. Through iterative coding, this study conducts content and cluster analysis to examine policy content. Findings show that while central government efforts promote coordination, local governments exhibit fragmented policy formation with inconsistent standards, imbalanced resource allocation, and disjointed governance. Three major policy challenges are identified: (1) data interoperability gaps due to a lack of national standards; (2) uneven regional funding distribution, with limited incentives for sustainable industry development; (3) overlapping stakeholders and redundant responsibilities, leading to regulatory inconsistencies. To mitigate fragmentation, we propose establishing an inter-ministerial coordination framework and strengthening digital infrastructure integration for nationwide interoperability in aged care services. As digital technologies are increasingly integrated into aging policies worldwide, ensuring policy coherence is crucial for effective governance. This study contributes to the discourse on technology-driven aging policy design and provides evidence-based insights for policy development.

